# (*Z*,1*S*,10a*R*)-(−)-Menthyl 1-hy­droxy-1,2,3,5,6,7,10,10a-octa­hydro­pyrrolo­[1,2-*a*]azocine-10a-carboxyl­ate

**DOI:** 10.1107/S1600536812027900

**Published:** 2012-06-27

**Authors:** Daniele Muroni, Emilio Napolitano, Olivier Perez, Nicola Culeddu, Antonio Saba

**Affiliations:** a Dipartimento di Chimica e Farmacia, Università degli Studi di Sassari, via Vienna 2, 07100 Sassari, Italy; b CRISMAT, UMR CNRS 6508, ENSICAEN, 6 Boulevard du Marechal Juin, F-14050 Caen CEDEX 4, France; c CNR Istituto di Chimica Biomolecolare sez. di Sassari, via La Crucca, Baldinca-Li Punti, 07040 Sassari, Italy

## Abstract

The structure determination confirms the stereochemistry of the title compound, C_21_H_35_NO_3_, obtained as an inter­mediate in the enanti­oselective synthesis of de­oxy­nojirimicine analogs. The system contains a pyrrolo­[1,2-*a*]azocine backbone, which was synthesized by a domino process involving a [2,3]-sigmatropic rearrangement. The incorporation of a chiral auxiliary (−)-menthyl, whose stereocentres are not involved during the synthesis, enables the assignation of absolute configuration. The crystal structure features O—H⋯O hydrogen bonds involving the hy­droxy groups as donors and the carbonyl groups as acceptors, which link the mol­ecules into chains running along [010].

## Related literature

For the construction of the pyrrolo­[1,2-*a*]azocine backbone by the domino sequence, see: Clark *et al.* (2001 ▶); Muroni *et al.* (2006 ▶). For domino processes promoted by catalytic decomposition of diazo­compounds, see: Doyle *et al.* (1997 ▶). For [2,3]-sigmatropic rearrangement, see: Sweeney (2009 ▶); Zhang & Wang (2010 ▶). For manzamine alkaloids and other biologically active compounds containing the pyrrolo­[1,2-*a*]azocine subunit, see: Rao *et al.* (2006 ▶); Yap *et al.* (2011 ▶); Sun *et al.* (2011 ▶). For de­oxy­nojirimicine and imino­sugars, see: Asano *et al.* (2000 ▶); Watson *et al.* (2001 ▶). For chiral auxiliary (−)-menthyl, see: Wang *et al.* (2006 ▶).
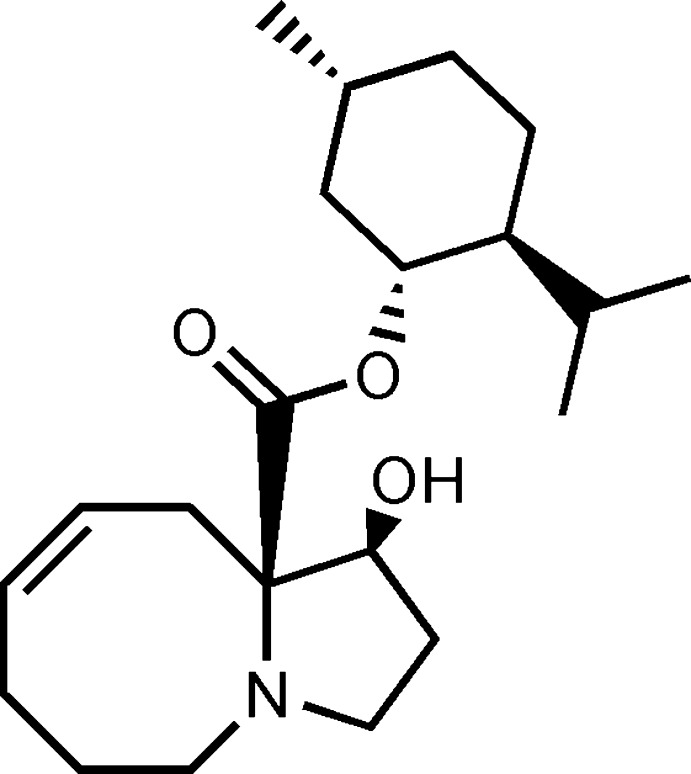


## Experimental

### 

#### Crystal data

C_21_H_35_NO_3_
*M* *_r_* = 349.5
Orthorhombic, 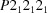

*a* = 10.7804 (8) Å
*b* = 7.7938 (7) Å
*c* = 23.8862 (17) Å
*V* = 2006.9 (3) Å^3^
*Z* = 4
Mo *K*α radiation
μ = 0.08 mm^−1^
*T* = 120 K
0.36 × 0.13 × 0.13 mm

#### Data collection

Bruker APEXII CCD diffractometer
Absorption correction: multi-scan (*SADABS*; Sheldrick, 2008 ▶) *T*_min_ = 0.716, *T*_max_ = 0.746
21709 measured reflections
3281 independent reflections
2331 reflections with *I* > 3σ(*I*)
*R*_int_ = 0.048

#### Refinement

*R*[*F* ^2^ > 2σ(*F* ^2^)] = 0.041
*wR*(*F* ^2^) = 0.047
*S* = 1.23
3281 reflections
230 parameters
H atoms treated by a mixture of independent and constrained refinement
Δρ_max_ = 0.23 e Å^−3^
Δρ_min_ = −0.28 e Å^−3^

### 

Data collection: *APEX2* (Bruker, 2005 ▶); cell refinement: *SAINT* (Bruker, 2007 ▶); data reduction: *SAINT*; program(s) used to solve structure: *SIR2011* (Burla *et al.*, 2012 ▶); program(s) used to refine structure: *JANA2006* (Petricek *et al.*, 2006 ▶); molecular graphics: *DIAMOND* (Brandenburg & Putz, 2005 ▶) and *ORTEP-3* (Farrugia, 1997 ▶); software used to prepare material for publication: *publCIF* (Westrip, 2010 ▶) and *PLATON* (Spek, 2009 ▶).

## Supplementary Material

Crystal structure: contains datablock(s) global, I. DOI: 10.1107/S1600536812027900/bh2433sup1.cif

Supplementary material file. DOI: 10.1107/S1600536812027900/bh2433Isup2.cdx

Structure factors: contains datablock(s) I. DOI: 10.1107/S1600536812027900/bh2433Isup3.hkl

Supplementary material file. DOI: 10.1107/S1600536812027900/bh2433Isup4.cml

Additional supplementary materials:  crystallographic information; 3D view; checkCIF report

## Figures and Tables

**Table 1:** Hydrogen-bond geometry (Å, °)

*D*—H⋯*A*	*D*—H	H⋯*A*	*D*⋯*A*	*D*—H⋯*A*
O1—H1*o*⋯O2^i^	0.81 (2)	2.02 (2)	2.8259 (19)	174 (2)

Symmetry code: (i) 
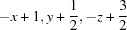
.

**Fig. 1.**
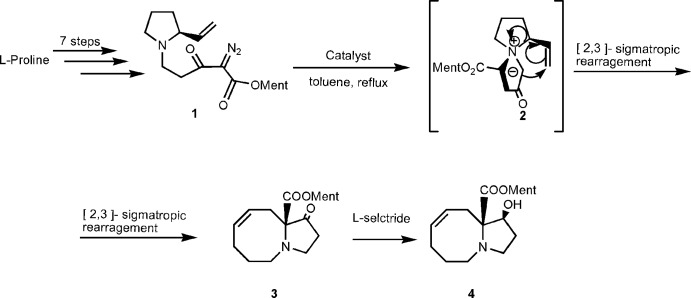
Synthesis scheme.

**Fig. 2.**
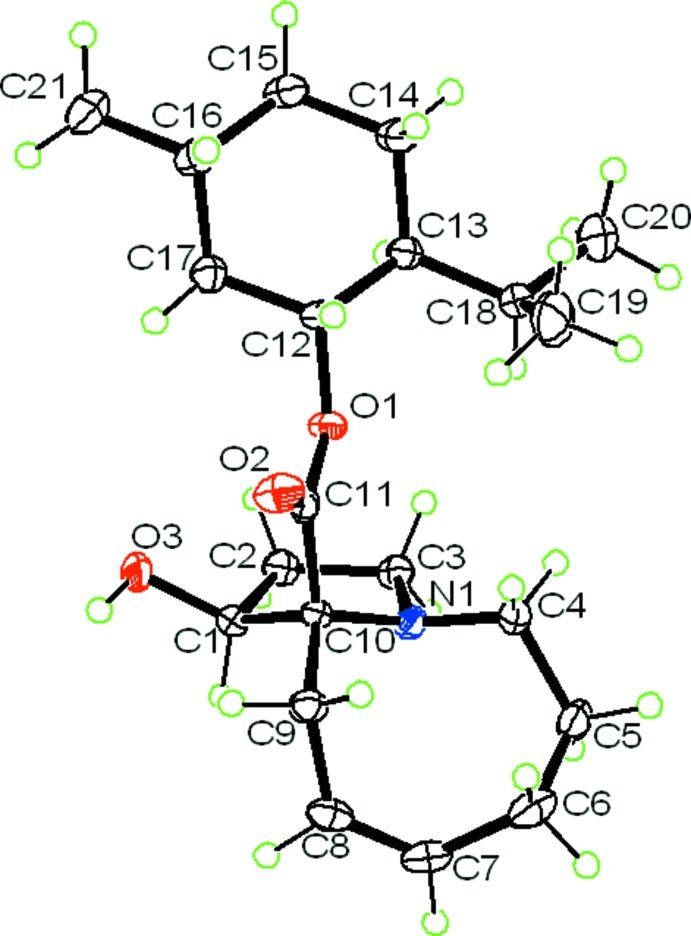
The molecular structure of the title compound, showing displacement ellipsoids at the 50% probability level.

**Fig. 3.**
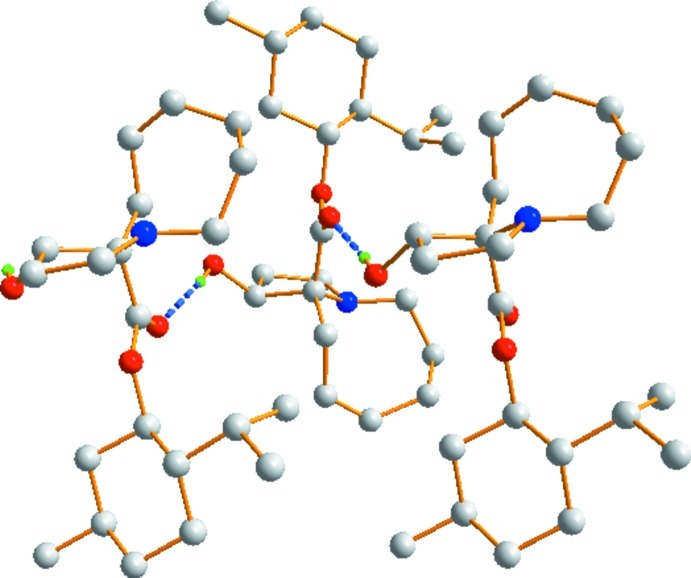
H-bridged molecule of title compound in the solid state. The The O—H···O hydrogen bonding is shown as blue dashed lines.

**Fig. 4.**
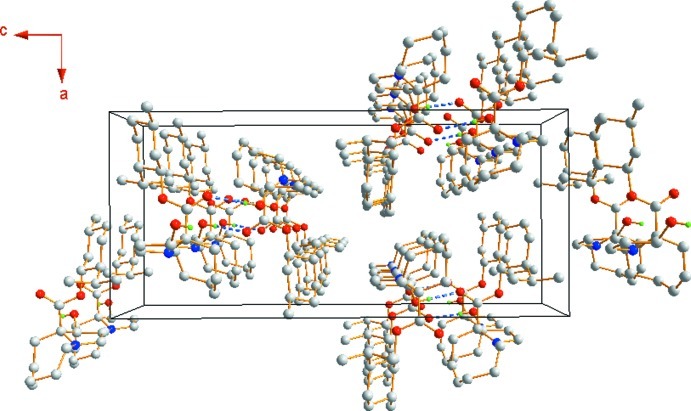
The crystal packing of the title compound viewed along the b axis. The H atoms not involved in intermolecular interactions (dashed lines) have been omitted for clarity.

## supplementary crystallographic information

### Comment

The pyrrolo[1,2-*a*]azocine backbone is contained as the *CE* subunit in the structures of manzamine and ircinal alkaloids (Rao *et al.*, 2006), as well as other natural and synthetic compounds which have shown interesting biological properties (Yap *et al.*, 2011; Sun *et al.*, 2011). Among the methods for a rapid construction of bicycle alkaloid, domino processes promoted by catalytic decomposition of diazo compounds have become commonly employed since they give a rapid access to complex structures in a stereoselective way (Doyle *et al.*, 1997; Clark *et al.*, 2001).

The used route for the synthesis of the title compound is described in Figure 1. The diazocarbonyl derivative 1 was synthesized starting from *L*-proline and (–)-menthyl acetate. The decomposition in refluxing toluene with Cu(acac)_2_ or Rh_2_(OAc)_4_ brought in one step to the pyrroloazocine alkaloid 3. The decomposition triggers a domino process that involved a carbenoidic attack to the nitrogen lone pair and the formation of the [5,5]-spirocyclic ammonium ylide 2. The ylide undergoes a [2,3]-sigmatropic rearrangement and it was possible to isolate the alkaloid 3 in 70% yield and 97% enantiomeric excess (Muroni *et al.*, 2006). The stereo specific nature of [2,3]-sigmatropic rearrangement allows a complete transfer of chirality (Sweeney, 2009; Zhang & Wang, 2010). As first step in the conversion in deoxynojirimicine analogs (Asano *et al.*, 2000; Watson *et al.*, 2001), the reduction of the carbonyl with *L*-selectride gave, after chromatography and recrystallization, the title compound 4 as a single diastereoisomer. The structure determination confirms the configuration of the quaternary stereocentre formed during the domino sequence and the configuration of the carbinol function, which is in accordance with the attack of *L*-selectride at the opposite face of the ester function.

The structural model (Fig. 2) showed standard bond lengths and angles; the X-ray analysis confirmed a *cis* C7═C8 double bond in the azocine ring, and the stereochemistry of C atoms known from literature in the auxiliary chiral (–)-menthyl: *S*-C13, *R*-C12 and *R*-C16. Two new chiral centers were identified *S*-C1 and *R*-C10.

The crystal structure (Fig. 3 and 4) consists of one type of O—H···O hydrogen-bond, with each molecule acting as a donor and acceptor of two hydrogen bonds. One molecule is linked through hydrogen interaction to other two symmetry-related molecules in the crystal, resulting in the formation of chains parallel to the [010] direction.

### Experimental

All ^1^H NMR (400 MHz) and ^13^C NMR (100 MHz) spectra were recorded on a Varian Mercury plus 400 spectrometer. Infrared (IR) spectra were performed on a FT/IR-480plus JASKO spectrophotometer. The optical rotations were measured by a polarimeter P-1010 JASCO in a 1 dm tube. All reagents and solvents employed were reagent grade materials purified by standard methods and redistilled before use. (1*R*)-(–)-menthyl acetate (>98%) and *L*-proline (>99.0%) were purchased from Sigma-Aldrich.

To a solution of compound 3 (210 mg, 0.6 mmol, see Fig. 1) in dry THF (5 ml) was added *L*-selectride (1.21 ml of 1.0 *M* solution in THF, 1.21 mmol) dropwise at 273 K. The reaction mixture was stirred for 1 h at 273 K and then allowed to warm to room temperature for another 1 h. The mixture was then diluted with EtOAc (50 ml) and filtered through a pad of silica gel, which was rinsed with EtOAc (50 ml). The filtrate was concentrated under reduced pressure, and the residue purified by flash chromatography (petroleum ether/ethyl acetate, 9:1) to give 180 mg of 4 (85%) as white oil. Recrystallization from EtOH/H_2_O (8:2) gave the title compound 4 as white crystals: m.p. 389 K; [α]^25^_D_ = -92.94 (*c* 0.32, CHCl_3_); ^1^H NMR (CDCl_3_): δ 0.74 (d, 3H, J=7.0 Hz), 0.89 (d, 3H, J=6.8 Hz), 0.91 (d, 3H, J=6.8 Hz), 0.80–1.11 (m, 3H), 1.30–1.58 (m, 3H), 1.60–1.73 (m, 3H), 1.73–1.86 (m, 1H), 1.92–2.10 (m, 3H), 2.12–2.32 (m, 3H), 2.37 (d, 1H, J=8.4 Hz), 2.64–2.80 (m, 2H), 2.92 (ddd, 1H, J=15.8, 12.1, 3.1 Hz), 3.00 (dt, 1H, J=9, 5.2 Hz), 3.15 (dt, 1H, J=8.4, 5.2 Hz), 3.98 (q, 1H, J=8.0 Hz), 4.74 (dt, 1H, J=4.4, 10.8 Hz), 5.65–5.80 p.p.m. (m, 2H). ^13^C NMR (CDCl_3_): δ 15.71, 20.90, 22.03, 22.91, 25.40, 25.89, 29.12, 31.39, 31.45, 32.50, 34.22, 41.18, 47.07 48.21, 50.04, 74.97, 75.08, 78.01, 126.17, 132.84, 173.64 p.p.m.; IR (neat): 3465, 3019, 2954, 1708, 1456, 1214 cm^-1^. Anal. Calc. for C_21_H_35_NO_3_: C 72.17, H 10.09, N 4.01%; Found: C 72.20, H 10.05, N 4.05%.

### Refinement

All C-bonded H atoms were fixed geometrically and treated as riding with C—H = 0.96 Å and with *U*_iso_(H) = 1.2*U*_eq_(carrier C). The hydroxyl H-atom H1o was located in a difference map, and included in the subsequent refinement with *U*_iso_(H1o) = 1.2*U*_eq_(O1). All H atoms were refined isotropically. The absolute configuration was assigned from the use of the chiral auxiliary (–)-menthyl (Wang *et al.*, 2006) as the starting material, whose stereo centres are not involved in the reaction. Owing to the absence of significant anomalous dispersion for data collected with the Mo radiation, 2332 measured Friedel pairs were merged.

### Crystal data

**Table:**

C_21_H_35_NO_3_	*D*_x_ = 1.156 Mg m^−^^3^
*M**_r_* = 349.5	Melting point: 389 K
Orthorhombic, *P*2_1_2_1_2_1_	Mo *K*α radiation, λ = 0.71069 Å
Hall symbol: P 2ac 2ab	Cell parameters from 192 reflections
*a* = 10.7804 (8) Å	θ = 3.8–17.2°
*b* = 7.7938 (7) Å	µ = 0.08 mm^−^^1^
*c* = 23.8862 (17) Å	*T* = 120 K
*V* = 2006.9 (3) Å^3^	Prism, colourless
*Z* = 4	0.36 × 0.13 × 0.13 mm
*F*(000) = 768	

### Data collection

**Table:**

Bruker APEXII CCD diffractometer	3281 independent reflections
Radiation source: sealed X-ray tube	2331 reflections with *I* > 3σ(*I*)
Graphite monochromator	*R*_int_ = 0.048
ω scans	θ_max_ = 30.0°, θ_min_ = 1.7°
Absorption correction: multi-scan (*SADABS*; Sheldrick, 2008)	*h* = −15→14
*T*_min_ = 0.716, *T*_max_ = 0.746	*k* = −10→4
21709 measured reflections	*l* = −33→33

### Refinement

**Table:**

Refinement on *F*	H atoms treated by a mixture of independent and constrained refinement
*R*[*F*^2^ > 2σ(*F*^2^)] = 0.041	Weighting scheme based on measured s.u.'s *w* = 1/(σ^2^(*F*) + 0.0004*F*^2^)
*wR*(*F*^2^) = 0.047	(Δ/σ)_max_ = 0.016
*S* = 1.23	Δρ_max_ = 0.23 e Å^−^^3^
3281 reflections	Δρ_min_ = −0.28 e Å^−^^3^
230 parameters	Extinction correction: B-C type 1 Gaussian isotropic
0 restraints	Extinction coefficient: 16900 (1800)
0 constraints	

### Fractional atomic coordinates and isotropic or equivalent isotropic displacement parameters (Å^2^)

**Table:**

	*x*	*y*	*z*	*U*_iso_*/*U*_eq_	
N1	0.67923 (4)	0.06933 (14)	0.867458 (19)	0.0178 (4)	
C4	0.67762 (4)	−0.11800 (4)	0.865956 (9)	0.0220 (5)	
C5	0.80174 (3)	−0.19770 (3)	0.849823 (11)	0.0257 (6)	
C6	0.83930 (2)	−0.17312 (6)	0.788428 (13)	0.0300 (6)	
C7	0.87823 (6)	0.0057 (2)	0.77279 (3)	0.0269 (6)	
C8	0.80364 (17)	0.13680 (12)	0.76224 (5)	0.0248 (6)	
C9	0.66451 (6)	0.12758 (8)	0.76547 (3)	0.0217 (5)	
C10	0.61339 (12)	0.16447 (13)	0.82487 (7)	0.0164 (5)	
C1	0.63656 (4)	0.35106 (14)	0.84420 (7)	0.0191 (5)	
O1	0.54447 (13)	0.46881 (18)	0.82643 (6)	0.0257 (4)	
C2	0.63894 (2)	0.34119 (7)	0.90814 (3)	0.0217 (5)	
C3	0.67015 (2)	0.15400 (4)	0.92186 (3)	0.0210 (5)	
C11	0.47217 (16)	0.1302 (2)	0.82373 (7)	0.0164 (5)	
O2	0.41170 (12)	0.10136 (18)	0.78189 (5)	0.0266 (4)	
O3	0.42289 (10)	0.13359 (17)	0.87513 (5)	0.0182 (4)	
C12	0.28911 (11)	0.1047 (2)	0.88055 (3)	0.0181 (5)	
C17	0.22301 (3)	0.27715 (11)	0.880303 (16)	0.0229 (6)	
C16	0.08230 (4)	0.24991 (5)	0.88606 (2)	0.0268 (6)	
C21	0.01309 (2)	0.42102 (2)	0.886679 (15)	0.0438 (8)	
C15	0.05454 (3)	0.14406 (3)	0.93799 (2)	0.0280 (6)	
C14	0.12697 (3)	−0.02306 (5)	0.940244 (13)	0.0253 (6)	
C13	0.26755 (15)	0.00578 (9)	0.93465 (2)	0.0188 (5)	
C18	0.34514 (5)	−0.16029 (4)	0.93837 (3)	0.0251 (6)	
C20	0.32327 (3)	−0.25601 (4)	0.993247 (7)	0.0414 (8)	
C19	0.32833 (4)	−0.27844 (2)	0.888694 (8)	0.0426 (8)	
H4a	0.614688	−0.156111	0.840384	0.0264*	
H4b	0.6521	−0.161351	0.901768	0.0264*	
H5a	0.800812	−0.317901	0.858591	0.0309*	
H5b	0.865733	−0.153207	0.873703	0.0309*	
H6b	0.904465	−0.252127	0.779173	0.036*	
H6a	0.772558	−0.209188	0.764565	0.036*	
H7a	0.965749	0.027044	0.77019	0.0323*	
H8a	0.840656	0.244258	0.75191	0.0298*	
H9a	0.637241	0.016292	0.753417	0.0261*	
H9b	0.629027	0.207304	0.739349	0.0261*	
H1a	0.712104	0.393489	0.828043	0.023*	
H2a	0.558255	0.368556	0.922615	0.0261*	
H2b	0.703241	0.414569	0.92227	0.0261*	
H3b	0.749001	0.148824	0.940459	0.0251*	
H3a	0.603436	0.104084	0.94292	0.0251*	
H12a	0.256529	0.038941	0.849922	0.0217*	
H17b	0.24025	0.335803	0.845829	0.0275*	
H17a	0.25235	0.34555	0.910993	0.0275*	
H16a	0.053248	0.1875	0.853987	0.0322*	
H21a	0.032739	0.48441	0.853379	0.0526*	
H21c	−0.074614	0.400151	0.888079	0.0526*	
H21b	0.037679	0.485998	0.91897	0.0526*	
H15b	−0.032695	0.11983	0.939646	0.0336*	
H15a	0.072245	0.210793	0.970814	0.0336*	
H14a	0.099119	−0.097775	0.910867	0.0303*	
H14b	0.10981	−0.080807	0.974889	0.0303*	
H13a	0.296357	0.071257	0.96611	0.0225*	
H18a	0.430002	−0.123046	0.937575	0.0302*	
H20a	0.33419	−0.178579	1.024112	0.0496*	
H20c	0.240371	−0.300948	0.993776	0.0496*	
H20b	0.381516	−0.348697	0.996362	0.0496*	
H19a	0.34236	−0.215556	0.854721	0.0512*	
H19c	0.386534	−0.371357	0.89113	0.0512*	
H19b	0.245414	−0.32338	0.88871	0.0512*	
H1o	0.556 (2)	0.499 (3)	0.7944 (9)	0.0309*	

### Atomic displacement parameters (Å^2^)

**Table:**

	*U*^11^	*U*^22^	*U*^33^	*U*^12^	*U*^13^	*U*^23^
N1	0.0178 (7)	0.0180 (8)	0.0174 (7)	0.0028 (6)	−0.0002 (6)	0.0006 (6)
C4	0.0200 (9)	0.0191 (10)	0.0268 (10)	0.0010 (7)	−0.0007 (8)	0.0041 (8)
C5	0.0218 (10)	0.0180 (10)	0.0374 (11)	0.0050 (7)	−0.0012 (8)	−0.0013 (9)
C6	0.0215 (10)	0.0320 (12)	0.0364 (11)	0.0038 (9)	0.0023 (9)	−0.0108 (10)
C7	0.0169 (9)	0.0376 (12)	0.0263 (10)	−0.0026 (8)	0.0056 (8)	−0.0079 (10)
C8	0.0239 (9)	0.0303 (11)	0.0203 (9)	−0.0050 (9)	0.0069 (8)	−0.0030 (9)
C9	0.0211 (9)	0.0252 (10)	0.0189 (9)	0.0016 (8)	0.0046 (7)	−0.0009 (8)
C10	0.0148 (8)	0.0183 (9)	0.0162 (8)	0.0005 (7)	0.0025 (7)	0.0033 (8)
C1	0.0180 (8)	0.0179 (9)	0.0215 (9)	0.0000 (7)	0.0040 (7)	0.0033 (8)
O1	0.0307 (7)	0.0224 (7)	0.0241 (7)	0.0078 (6)	0.0055 (6)	0.0083 (6)
C2	0.0214 (9)	0.0205 (10)	0.0232 (9)	−0.0018 (8)	0.0017 (7)	−0.0020 (8)
C3	0.0173 (9)	0.0263 (10)	0.0193 (9)	0.0007 (8)	−0.0017 (7)	0.0004 (8)
C11	0.0193 (8)	0.0142 (9)	0.0159 (8)	0.0040 (7)	0.0016 (7)	0.0014 (8)
O2	0.0197 (6)	0.0408 (9)	0.0192 (7)	0.0014 (6)	−0.0014 (5)	−0.0028 (6)
O3	0.0139 (6)	0.0243 (7)	0.0164 (6)	−0.0016 (5)	0.0022 (5)	0.0009 (6)
C12	0.0132 (8)	0.0227 (10)	0.0184 (9)	−0.0017 (7)	0.0015 (6)	−0.0005 (8)
C17	0.0226 (9)	0.0239 (11)	0.0223 (10)	0.0032 (8)	0.0007 (8)	0.0037 (8)
C16	0.0189 (9)	0.0340 (12)	0.0276 (11)	0.0040 (9)	−0.0018 (8)	0.0000 (9)
C21	0.0283 (11)	0.0506 (16)	0.0527 (15)	0.0167 (11)	0.0056 (10)	0.0170 (13)
C15	0.0183 (9)	0.0321 (12)	0.0336 (11)	0.0022 (9)	0.0067 (8)	−0.0013 (10)
C14	0.0189 (9)	0.0251 (11)	0.0318 (11)	−0.0023 (8)	0.0052 (8)	0.0000 (9)
C13	0.0177 (8)	0.0202 (10)	0.0185 (9)	−0.0009 (7)	0.0014 (7)	−0.0001 (8)
C18	0.0202 (9)	0.0203 (10)	0.0348 (11)	0.0020 (8)	0.0071 (8)	0.0055 (9)
C20	0.0396 (13)	0.0359 (13)	0.0485 (13)	0.0092 (11)	0.0061 (11)	0.0186 (11)
C19	0.0566 (16)	0.0222 (12)	0.0492 (14)	0.0067 (11)	0.0135 (12)	−0.0031 (10)

### Geometric parameters (Å, º)

**Table:**

N1—C4	1.4605 (11)	C11—O3	1.338 (2)
N1—C10	1.4450 (16)	O3—C12	1.4654 (16)
N1—C3	1.4607 (9)	C12—C17	1.5209 (17)
C4—C5	1.5247 (5)	C12—C13	1.5228 (11)
C4—H4a	0.9600	C12—H12a	0.9600
C4—H4b	0.9600	C17—C16	1.5379 (6)
C5—C6	1.5335 (4)	C17—H17b	0.9600
C5—H5a	0.9600	C17—H17a	0.9600
C5—H5b	0.9600	C16—C21	1.5282 (4)
C6—C7	1.5029 (18)	C16—C15	1.5195 (7)
C6—H6b	0.9600	C16—H16a	0.9600
C6—H6a	0.9600	C21—H21a	0.9600
C7—C8	1.324 (2)	C21—H21c	0.9600
C7—H7a	0.9600	C21—H21b	0.9600
C8—C9	1.5035 (19)	C15—C14	1.5196 (5)
C8—H8a	0.9600	C15—H15b	0.9600
C9—C10	1.5492 (17)	C15—H15a	0.9600
C9—H9a	0.9600	C14—C13	1.5379 (16)
C9—H9b	0.9600	C14—H14a	0.9600
C10—C1	1.5461 (16)	C14—H14b	0.9600
C10—C11	1.546 (2)	C13—C18	1.5436 (11)
C1—O1	1.4169 (17)	C13—H13a	0.9600
C1—C2	1.5294 (18)	C18—C20	1.5266 (6)
C1—H1a	0.9600	C18—C19	1.5128 (6)
O1—H1o	0.81 (2)	C18—H18a	0.9600
C2—C3	1.5327 (6)	C20—H20a	0.9600
C2—H2a	0.9600	C20—H20c	0.9600
C2—H2b	0.9600	C20—H20b	0.9600
C3—H3b	0.9600	C19—H19a	0.9600
C3—H3a	0.9600	C19—H19c	0.9600
C11—O2	1.214 (2)	C19—H19b	0.9600
			
C4—N1—C10	119.33 (6)	C11—O3—C12	117.97 (11)
C4—N1—C3	118.20 (5)	O3—C12—C17	108.98 (11)
C10—N1—C3	111.19 (8)	O3—C12—C13	107.64 (10)
N1—C4—C5	113.76 (3)	O3—C12—H12a	112.00
N1—C4—H4a	109.47	C17—C12—C13	112.28 (7)
N1—C4—H4b	109.47	C17—C12—H12a	107.00
C5—C4—H4a	109.47	C13—C12—H12a	109.00
C5—C4—H4b	109.47	C12—C17—C16	109.87 (7)
H4a—C4—H4b	105.00	C12—C17—H17b	109.47
C4—C5—C6	115.00 (2)	C12—C17—H17a	109.47
C4—C5—H5a	109.47	C16—C17—H17b	109.47
C4—C5—H5b	109.47	C16—C17—H17a	109.47
C6—C5—H5a	109.47	H17b—C17—H17a	109.00
C6—C5—H5b	109.47	C17—C16—C21	111.22 (4)
H5a—C5—H5b	103.00	C17—C16—C15	110.02 (4)
C5—C6—C7	115.30 (4)	C17—C16—H16a	109.00
C5—C6—H6b	109.47	C21—C16—C15	111.69 (4)
C5—C6—H6a	109.47	C21—C16—H16a	107.00
C7—C6—H6b	109.47	C15—C16—H16a	108.00
C7—C6—H6a	109.47	C16—C21—H21a	109.47
H6b—C6—H6a	103.00	C16—C21—H21c	109.47
C6—C7—C8	126.38 (9)	C16—C21—H21b	109.47
C6—C7—H7a	117.00	H21a—C21—H21c	109.00
C8—C7—H7a	117.00	H21a—C21—H21b	109.00
C7—C8—C9	124.00 (10)	H21c—C21—H21b	109.47
C7—C8—H8a	118.00	C16—C15—C14	113.14 (3)
C9—C8—H8a	118.00	C16—C15—H15b	109.00
C8—C9—C10	113.14 (8)	C16—C15—H15a	109.47
C8—C9—H9a	109.47	C14—C15—H15b	109.47
C8—C9—H9b	109.47	C14—C15—H15a	109.47
C10—C9—H9a	109.47	H15b—C15—H15a	106.00
C10—C9—H9b	109.47	C15—C14—C13	112.21 (4)
H9a—C9—H9b	105.53	C15—C14—H14a	109.47
N1—C10—C9	112.01 (8)	C15—C14—H14b	109.47
N1—C10—C1	101.14 (11)	C13—C14—H14a	109.47
N1—C10—C11	114.03 (11)	C13—C14—H14b	109.47
C9—C10—C1	112.99 (10)	H14a—C14—H14b	107.00
C9—C10—C11	107.60 (11)	C12—C13—C14	107.36 (9)
C1—C10—C11	109.07 (10)	C12—C13—C18	113.00 (9)
C10—C1—O1	114.00 (10)	C12—C13—H13a	110.00
C10—C1—C2	104.69 (10)	C14—C13—C18	113.97 (6)
C10—C1—H1a	110.00	C14—C13—H13a	109.00
O1—C1—C2	110.07 (10)	C18—C13—H13a	103.00
O1—C1—H1a	105.00	C13—C18—C20	112.05 (5)
C2—C1—H1a	114.00	C13—C18—C19	113.60 (5)
C1—O1—H1o	111.1 (16)	C13—C18—H18a	105.00
C1—C2—C3	105.37 (6)	C20—C18—C19	110.95 (2)
C1—C2—H2a	109.47	C20—C18—H18a	108.00
C1—C2—H2b	109.47	C19—C18—H18a	106.00
C3—C2—H2a	109.47	C18—C20—H20a	109.47
C3—C2—H2b	109.47	C18—C20—H20c	109.47
H2a—C2—H2b	113.00	C18—C20—H20b	109.47
N1—C3—C2	104.74 (6)	H20a—C20—H20c	109.47
N1—C3—H3b	109.47	H20a—C20—H20b	109.47
N1—C3—H3a	109.47	H20c—C20—H20b	109.47
C2—C3—H3b	109.47	C18—C19—H19a	109.47
C2—C3—H3a	109.47	C18—C19—H19c	109.47
H3b—C3—H3a	114.00	C18—C19—H19b	109.47
C10—C11—O2	125.10 (15)	H19a—C19—H19c	109.47
C10—C11—O3	111.82 (14)	H19a—C19—H19b	109.47
O2—C11—O3	123.07 (16)	H19c—C19—H19b	109.00

### Hydrogen-bond geometry (Å, º)

**Table:**

*D*—H···*A*	*D*—H	H···*A*	*D*···*A*	*D*—H···*A*
O1—H1*o*···O2^i^	0.81 (2)	2.02 (2)	2.8259 (19)	174 (2)

Symmetry code: (i) −*x*+1, *y*+1/2, −*z*+3/2.
